# Stability and Change of Psychopathology Symptoms Throughout Childhood and Adolescence

**DOI:** 10.1007/s10578-021-01212-8

**Published:** 2021-06-28

**Authors:** Elisabet Blok, C. Louk de Mol, Jan van der Ende, Manon H. J. Hillegers, Robert R. Althoff, Philip Shaw, Tonya White

**Affiliations:** 1 Department of Child and Adolescent Psychiatry/Psychology, Erasmus MC-Sophia Children’s Hospital, Kamer KP-2869, Postbus 2060, 3000 CB Rotterdam, The Netherlands; 2 Generation R Study Group, Erasmus MC, Rotterdam, The Netherlands; 3 Department of Neurology, MS Center ErasMS, Erasmus MC, Rotterdam, The Netherlands; 4 Department of Psychiatry, University Vermont, Burlington, USA; 5 Office of the Clinical Director, National Institute of Mental Health, Bethesda, MD 20892 USA; 6 Neurobehavioral Clinical Research Section, Social and Behavioral Research Branch, National Human Genome Research Institute, Bldg 31 B137, Bethesda, 20892 USA; 7 Department of Radiology and Nuclear Medicine, Erasmus MC, Rotterdam, The Netherlands

**Keywords:** Psychopathology, Development, Child Behavior Checklist (CBCL), Latent transition analysis (LTA)

## Abstract

Assessing stability and change of children’s psychopathology symptoms can help elucidate whether specific behaviors are transient developmental variations or indicate persistent psychopathology. This study included 6930 children across early childhood (T1), late childhood (T2) and early adolescence (T3), from the general population. Latent profile analysis identified psychopathology subgroups and latent transition analysis quantified the probability that children remained within, or transitioned across psychopathology subgroups. We identified four psychopathology subgroups; no problems (T1: 85.9%, T2: 79.0%, T3: 78.0%), internalizing (T1: 5.1%, T2: 9.2%, T3: 9.0%), externalizing (T1: 7.3%, T2: 8.3%, T3: 10.2%) and the dysregulation profile (DP) (T1: 1.7%, T2: 3.5%, T3: 2.8%). From T1 to T2, 44.7% of the children remained in the DP. Between T2 and T3, 33.6% remained in the DP; however, 91.4% were classified in one of the psychopathology subgroups. Our findings suggest that for many children, internalizing or externalizing symptoms encompass a transient phase within development. Contrary, the DP resembles a severe at-risk state in which the predictive value for being in one of the psychopathology subgroups increases over time.

**Supplementary Information:**

The online version contains supplementary material available at 10.1007/s10578-021-01212-8.

## Introduction

It is widely known that children with symptoms of psychopathology in childhood and adolescence are at higher risk of psychopathology in adulthood [1–5]. Studying the stability and change of child behavior over time can elucidate those characteristics of psychopathology that are transient phases of development versus those that signal persistent psychopathology. Symptoms of psychopathology in children can be divided broadly into two domains; namely internalizing (e.g. anxiety) and externalizing problems (e.g. aggressive behavior). Additionally, there is a group of children with comorbid internalizing and externalizing problems. In the literature these children have been labelled as either a comorbid group or a group with the dysregulation profile (DP) [5–8].

Many studies have assessed the prevalence of psychopathology and the continuity of individual domains of psychopathology separately (e.g. depressive symptoms, aggressive behavior) [1, 9–11]. These studies provide evidence that overall stability decreases when the time interval between the first measurement and follow-up waves increases [9–11], and that externalizing behavior tends to be more stable than internalizing behavior [9]. Additionally, stability in symptoms of psychopathology has been shown to be higher in children who were older at initial assessment, suggesting that symptoms of psychopathology become more predictive of persistent psychopathology with age [1]. However, studying persistence and change of individual domains of psychopathology has two limitations. First, as internalizing and externalizing symptoms are highly comorbid [10], the continuity and change of the correlated domains will likely, to some extent, reflect the same underlying process. Second, when there is a reduction of symptoms in one domain, we do not know whether this reflects a decrease in overall symptoms, or alternatively, a change only in those specific symptoms (e.g. decrease in aggression coupled with an increase in attention problems).

An increasingly adopted method that identifies subgroups of children based on their continuous symptoms across domains of psychopathology is latent profile analysis (LPA). This data-driven approach minimizes the heterogeneity of comorbid symptoms and allows for a more integrated analysis of child behavior compared to assessing specific domains (e.g. depressive symptoms) separately. In a recent review, 23 studies that used LPA to study symptoms of psychopathology in 4–11 year-old children were compared [12]. Three of these studies analyzed symptoms across internalizing and externalizing domains in a population-based setting, of which two were performed at younger ages in the current sample [6, 13]. All studies identified four psychopathology subgroups; (i.) no problems, (ii.) internalizing, (iii.) externalizing and (iv.) a comorbid or DP group [6, 7, 13]. Across all ages, approximately 2% of the population was classified in the DP with comorbid internalizing and externalizing problems. The percentage of children included in either the internalizing or externalizing subgroup differed substantially between the two studies, with prior work in early childhood showing higher rates of externalizing, but lower rates of internalizing symptoms compared to studies in later childhood and adolescence [6, 7]. An internalizing subgroup was present in toddlers aged 3 to 4.5 years and children between 5 and 9 years of age, with 4.8% and 5.3% being included in the internalizing subgroup in these age ranges, respectively. An externalizing subgroup was present as early as 1.5 to 2.5 years of age, with 11.1% of the children exhibiting this profile. Further, between 3 and 4.5 years of age, 6.5% was included in the externalizing profile and between 5 and 9 years of age, the proportion of children included was 7.3% [6]. Within another sample, assessing children in late childhood (mean age 7.5 years) and early adolescence (mean age 14 years), respectively 16.1% and 13.9% of the children were included in the internalizing and 2.5% and 4.4% in the externalizing subgroup [7].

To answer questions on individual developmental trajectories within or across psychopathology subgroups, latent transition analysis (LTA) can be applied. During development, children can either remain in the same subgroup over time, or transition between psychopathology subgroups. The former is typically referred to as homotypic continuity, which elucidates the predictive validity of children remaining in the same subgroup at each subsequent time point. Transitioning between psychopathology subgroups is referred to as heterotypic continuity, in which an earlier psychopathology subgroup can be an underlying risk factor for a child to transition to a different subgroup. Earlier work on the stability of symptoms of psychopathology in children has shown that stability increases with age [6] and that between mid-childhood and early adolescence, homotypic continuity is higher for the internalizing and externalizing subgroups than for the DP [7]. More specifically, from those children included in the internalizing subgroup in late toddlerhood, only 23% remained in this subgroup in early childhood [6] and from those identified with internalizing symptoms in late childhood, 61% exhibited internalizing symptoms in early adolescence [7]. Likewise for the externalizing subgroup, homotypic continuity was present for 31% between early and late toddlerhood, 39% between late toddlerhood and early childhood [6] and 62% between late childhood and early adolescence [7]. Regarding the DP, homotypic continuity did not consistently increase across age. Between early and late toddlerhood, late toddlerhood and early childhood, and finally between late childhood and early adolescence, homotypic continuity of the DP was present for 37%, 18% and 44% [6, 7].

Our study addresses several knowledge gaps to further improve the understanding of stability and change in symptoms of psychopathology from childhood into adolescence. First, prior work by Basten et al. within the current sample, assessed the stability and change of psychopathology symptoms until early childhood [6], not yet covering late childhood and early adolescence. Studying the stability and change of psychopathology symptoms in this age period is crucial, given the rapid changes in behavior that occur during adolescence [14] and since many psychiatric disorders emerge during adolescence [15, 16]. Second, despite the fact that the two previous longitudinal studies applied similar analyses, they used different measures of psychopathology. Basten et al. used parent reported questionnaire data, assessing psychopathology as symptoms along a continuum, whereas McElroy et al. used a structured clinical interview, implemented as a parent reported questionnaire, generating binary variables for the presence of psychiatric diagnoses [6, 7]. Thus, it remains unclear whether the substantial differences in prevalence can be attributed to age differences or to differences in sample or methodology.

Within this context, it is the primary goal of this study to assess developmental trajectories of children with psychopathology symptoms as they progress into adolescence. We evaluate which psychopathology subgroups are present in the general population at different ages and the probability that individuals remain within or transition between the observed psychopathology subgroups over time. Based on previous literature, we hypothesized that we would observe four psychopathology subgroups (no problems, internalizing, externalizing and DP) in all age groups, and that the psychopathology subgroups become more stable with age, except for the DP group. We hypothesize that this group will begin to diverge during adolescence, transitioning to primarily either internalizing or externalizing subgroups.

## Method

### Participants

**Fig. 1:**
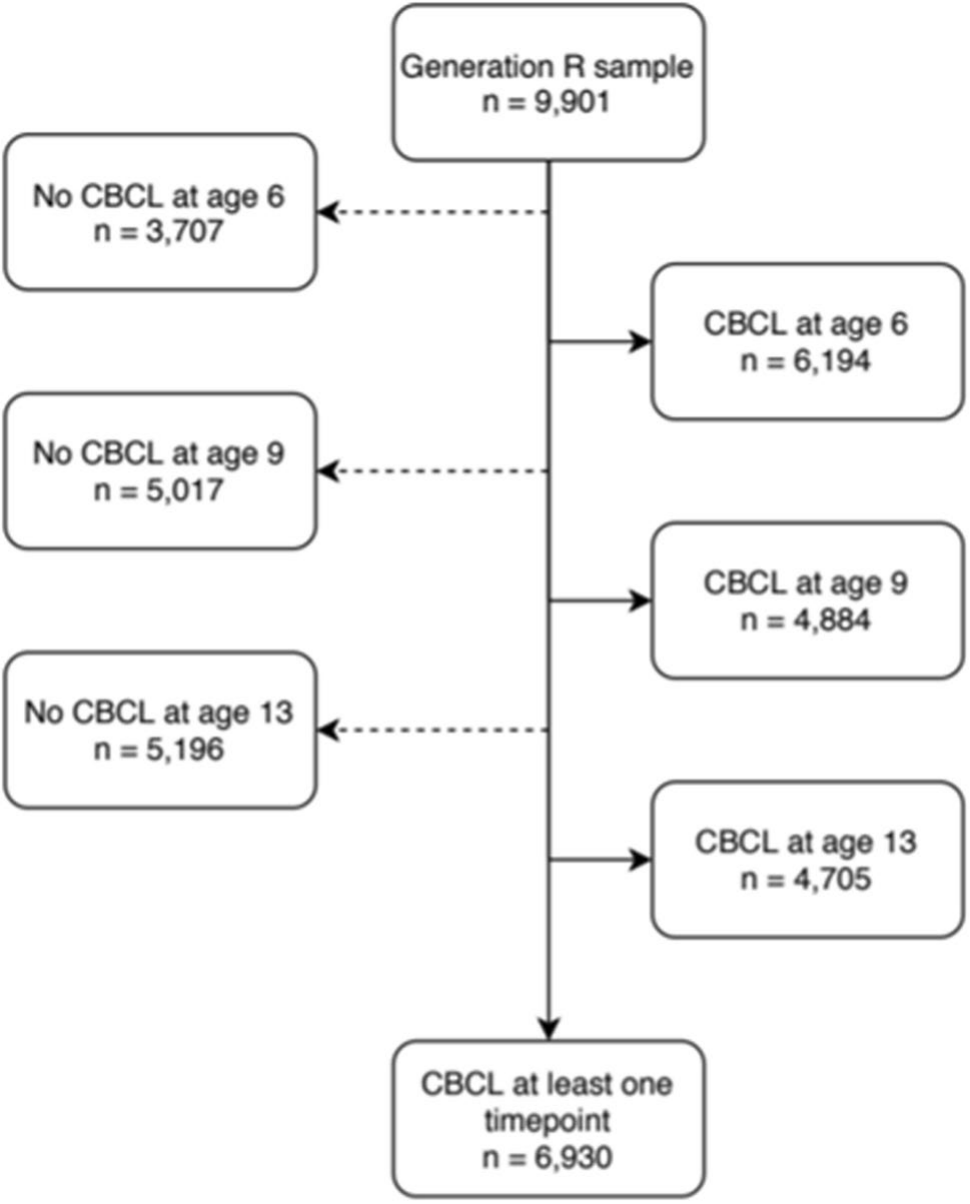
Flowchart of study sample

This study was embedded within the Generation R Study, which is a large longitudinal birth cohort in Rotterdam, the Netherlands, in which pregnant women with a delivery date between April 2002 and January 2006 were invited to participate [17]. Since recruitment, the children and their families have been invited for multiple waves of data collection. Children were included in this study if their parents filled out the Child Behavior Checklist (CBCL) at 5 to 8 (T1, n = 6194), at 9 to 12 (T2, n = 4884), or at 13 to 16 (T3, n = 4705) years of age. This resulted in a total sample of 6,930 participants who were eligible for inclusion. Of these participants, 736 had missing data at T1, 2046 had missing data at T2 and 2225 had missing data at T3. The majority of participants (n = 3633) participated in all measurement waves, 1587 participated in two measurement waves and 1710 participated in one measurement wave. Demographic information is provided in Table 1 and a flowchart of the study sample is provided in Fig. 1. The study was approved by the Medical Ethical Committee of the Erasmus Medical Centre in Rotterdam and was conducted according to the Declaration of Helsinki. Written informed consent was obtained from the legal representatives and, when children were older than 12 years-of-age, children also provided informed assent.

**Table 1:** Demographic characteristics

	Age 6 (n = 6194)	Age 9 (n = 4884)	Age 13 (n = 4705)
Age (M, SD)	6.06 (0.47)	9.71 (0.31)	13.55 (0.39)
Sex (% female)	49.6%	50.4%	50.4%
National origin (%)			
Western	71.0%	74.2%	74.2%
Non Western	28.9%	24.6%	24.8%
Missing	0.1%	1.1%	1.0%
Education (%)			
Low	6.6%	4.6%	5.1%
Middle	38.4%	37.6%	36.2%
High	48.4%	51.9%	52.9%
Missing	6.6%	5.9%	5.9%
Family income (%)			
< €2000 per month	21.3%	15.5%	14.2%
> €2000 per month	72.1%	70.8%	71.2%
Missing	6.7%	13.7%	14.6%

### Instruments

#### Child Behavior

Child behavior was assessed using the Child Behavior Checklist (CBCL). The CBCL version 1.5–5 years consists of 99 items, with a three-point Likert scale (0 = not true, 1 = somewhat true, 2 = very true). Seven empirically derived syndrome scales were calculated, being Anxious/Depressed, Aggressive Behavior, Emotionally Reactive, Somatic Complaints, Withdrawn, Sleep Problems and Attention Problems. Similarly, the CBCL version 6–18 years consists of 112 items, of which eight syndrome scales were derived, Anxious/Depressed, Withdrawn/Depressed, Somatic Complaints, Thought Problems, Attention Problems, Rule-breaking Behavior and Aggressive Behavior. Both versions are reliable and valid questionnaires for assessing behavioral problems [18, 19]. The primary caregiver completed the questionnaires, which was the CBCL v1.5-5 at T1 and CBCL v6-18 at T2 and T3 [20, 21]. Notably, given the age range included at T1, for some children the CBCL v1.5-5 and for other children the CBCL v6-18 would have been age appropriate. The CBCL v1.5-5 was used at T1 for all participants to keep the CBCL version consistent with earlier measurement waves of Generation R [20, 21]. T-scores of all syndrome scales were computed based on normative data for both the CBCL/1.5-5 and the CBCL/6-18 using ASEBA-PC [22].

#### Demographic Characteristics

Sex was obtained from birth records. Maternal education and household income were assessed through questionnaire. Maternal education was categorized into low (no education, primary school), middle (high school, vocational training) and high (higher vocational training, university). Household income was scored low in Generation R when parents had less than 2000 euros a month and high when parents had more than 2000 euros a month. Child national origin was based on the birth country of the parents and categorized as western (Dutch, American western, Asian western, European, Indonesian & Oceania) and non-western (African, American non-western, Asian non-western, Cape Verdean, Dutch Antilles, Moroccan, Surinamese & Turkish).

### Statistical Analyses

#### Latent Profile Analysis (LPA)

Psychopathology profiles at T1 were previously derived using the same procedures described below [13]. In earlier work, the Sleep Problems syndrome scale of the CBCL was not included in the analyses. Here, we applied LPA to define psychopathology subgroups at T1, T2 and T3, using CBCL T-scores of all available syndrome scales as indicators, including Sleep Problems. Consistent with earlier work within Generation R, we used five criteria to determine the optimal number of profiles [6, 13]. The Bayesian Information Criterion (BIC) and the bootstrapped likelihood ratio test (BLRT) were used to evaluate the fit of each model. The BIC is a measure of model fit considering the rule of parsimony, with a lower BIC indicating a better fit. The BLRT is a likelihood ratio for k classes versus k − 1 classes, with a p-value greater than 0.05 indicating that k − 1 classes are sufficient. Entropy of the models was evaluated with values closer to 1 indicating better classification. Moreover, all profiles obtained should include a minimum of 1% of the participants. Lastly, all profiles were inspected visually to ensure each additional profile had a distinct severity or shape pattern.

#### Latent Transition Analysis (LTA)

We applied LTA to calculate transition probabilities between psychopathology profiles over time. Due to the use of different CBCL versions (v1.5-5 at T1), we were unable to test a model where we held the profiles equal across all ages. Therefore, the quantitative change in profile patterns was taken into account in interpreting the transition probabilities. In our primary model we allowed profiles to be estimated freely. Additionally, a partial invariant model was tested in which the profiles were held equal between T2 and T3. Lastly, we tested a model in which demographic characteristics (biological sex, national origin and SES based on maternal education and household income) were included as covariates. The number of profiles included in the LTA at each age wave was based on the optimal number of profiles at each individual time point.

Both LPA and LTA were performed using Mplus version 8.6 [23]. Full information maximum likelihood (FIML) was used in both the LPA and the LTA analyses to account for missing data, which can be used to account for data considered either missing at random (MAR) or missing completely at random (MCAR). No missing data was imputed. A detailed overview showing in how many data collection waves each individual participated, split by demographic variables, is provided in Supplementary Table 1. Participants with a lower SES and participants from non-western national origin were more likely to have fewer measurements available, pointing towards a pattern of MAR.

## Results

### Latent Profile Analysis

The optimal model fit was determined, based on the BLRT, the BIC, the entropy and the inclusion of at least 1% of the participants in the smallest profile obtained. When two or more models fitted the data equally well, optimal fit was based on visual inspection. We were unable to discriminate between models based on the BLRT, since the BLRT was < 0.001 across all waves and across all tested profiles, indicating that adding a profile would improve model fit, even when six profiles were fitted to the data. Likewise, the BIC decreased with an increasing number of profiles added to the model and could therefore not discriminate between the models at T1–T3. At T1, a four-profile fit was determined the best fitting model, as the five-profile fit resulted in one profile with fewer than 1% of participants. At T2, the entropy varied between the models, indicating that the model with four, five or six profiles at T2 fit the data equally well. Visual inspection of the four and five profile fit showed that the fifth profile did not differ largely in both shape and severity from profiles that were already identified in the four-profile fit (Supplementary Fig. 1). Therefore, a four-profile fit was determined optimal at T2. At T3, the optimal fit was based on the entropy, which was highest for the four-profile fit. Model fit indices are provided in Supplementary Table 2.

**Fig. 2:**
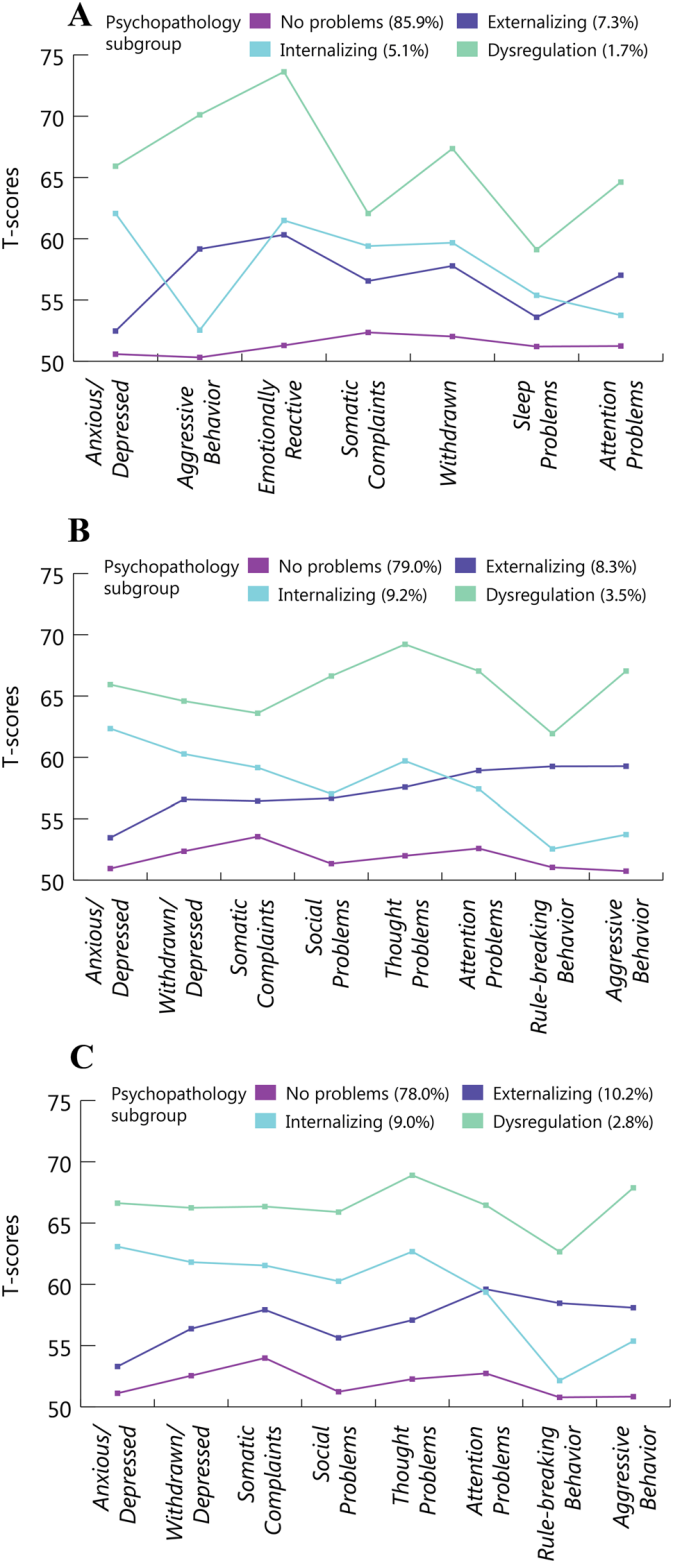
Behavioral subgroups derived with latent profile analyses **A** at T1, **B** at T2 and **C** at T3

We describe these four profiles as: (1.) no problems (T1: 85.9%, T2: 79.0%, T3: 78.0%) with low scores on all syndrome scales, (2.) internalizing (T1: 5.1%, T2: 9.2%, T3: 9.0%) with particularly high scores on Anxious/Depressed, Withdrawn/Depressed and Thought Problems, (3.) externalizing (T1: 7.3%, T2: 8.3%, T3: 10.2%), consisting of children scoring high on Attention Problems, Rule-Breaking Behavior and Aggressive Behavior, and finally (4.) the DP (T1: 1.7%, T2: 3.5%, T3: 2.8%), with elevated scores on all syndrome scales, see Table 2 and Fig. 2. To remain consistent with our earlier work, we use the term DP with the understanding that the term is synonymous with the term ‘comorbid group’.

**Table 2:** Mean T-scores of psychopathology profiles derived using LPA

Wave	Syndrome scale	Psychopathology subgroup			
		No problems	Internalizing	Externalizing	Dysregulation
T1	Anxious/Depressed	50.58	62.06	52.47	65.92
	Aggressive Behavior	50.31	52.54	59.16	70.11
	Emotionally reactive	51.29	61.49	60.32	73.62
	Somatic Complaints	52.35	59.40	56.55	62.06
	Withdrawn	52.02	59.67	57.78	67.35
	Sleep Problems	51.20	55.38	53.59	59.11
	Attention Problems	51.24	53.75	57.02	64.62
T2	Anxious/Depressed	50.95	62.34	53.45	65.93
	Withdrawn/Depressed	52.36	60.27	56.56	64.59
	Somatic Complaints	53.55	59.16	56.43	63.60
	Social Problems	51.35	57.02	56.66	66.63
	Thought Problems	51.99	59.70	57.58	69.22
	Attention Problems	52.58	57.42	58.92	67.04
	Rule-Breaking Behavior	51.04	52.55	59.26	61.93
	Aggressive Behavior	50.74	53.71	59.27	67.04
T3	Anxious/Depressed	51.11	63.08	53.29	66.63
	Withdrawn/Depressed	52.54	61.81	56.38	66.25
	Somatic Complaints	53.99	61.54	57.92	66.35
	Social Problems	51.23	60.26	55.63	65.90
	Thought Problems	52.27	62.67	57.08	68.90
	Attention Problems	52.73	59.36	59.60	66.46
	Rule-Breaking Behavior	50.78	52.14	58.46	62.66
	Aggressive Behavior	50.84	55.37	58.10	67.88

### Latent Transition Analysis

Based on fit statistics, the freely estimated model and the partial invariant model fitted the data equally well (freely estimated: BIC = 660,642.04, entropy = 0.87; partial invariant: BIC = 660,641.32, entropy = 0.87). Transition probabilities, based on the freely estimated model, between T1-T3 are shown in Tables 3 and 4. Transition probabilities for the partial invariant model are presented in Supplementary Table 3 and 4.

**Table 3:** Stability of psychopathology from T1 to T2

	No problems	Internalizing	Externalizing	Dysregulation
No problems	**0.845**	0.076	0.065	0.014
Internalizing	0.431	**0.381**	0.034	0.155
Externalizing	0.356	0.101	**0.373**	0.17
Dysregulation	0.219	0.111	0.223	**0.447**

^1^Profiles on the x-axis represent T2, profiles on the y-axis represent T1

^2^Profiles were not held equal over time, due to the fact that different versions of the CBCL were used (at T1: CBCL 1.5–5, at T2 and T3: CBCL 6–18)

^3^Bold numbers indicate homotypic continuity

^4^Model accounted for missing data using full information maximum likelihood (FIML) in Mplus

**Table 4:** Stability of psychopathology from T2 to T3

	No problems	Internalizing	Externalizing	Dysregulation
No problems	**0.881**	0.051	0.061	0.007
Internalizing	0.51	**0.376**	0.077	0.037
Externalizing	0.334	0.081	**0.468**	0.107
Dysregulation	0.086	0.356	0.221	**0.336**

^1^Profiles on the x-axis represent T3, profiles on the y-axis represent T2

^2^Profiles were not held equal over time, due to the fact that different versions of the CBCL were used (at T1: CBCL 1.5–5, at T2 and T3: CBCL 6–18)

^3^Bold numbers indicate homotypic continuity

^4^Model accounted for missing data using full information maximum likelihood (FIML) in Mplus

Homotypic continuity, indicating that children were classified in the same psychopathology subgroup at two consecutive time points, was 84.5% between T1 and T2 for the no problems group and 88.1% between T2 and T3. As expected, homotypic continuity was lower for the psychopathology subgroups than for the no problems group. Homotypic continuity between T1 and T2 and between T2 and T3 for the internalizing group was 38.1% and 37.6%, for the externalizing subgroup was 37.3% and 46.8%, and for the DP subgroup was 44.7% and 33.6%, respectively. Heterotypic continuity was present for all profiles, with some children transitioning from the no problems subgroup to internalizing (T1–T2: 7.6%, T2–T3: 5.1%) and externalizing (T1–T2: 6.5%, T2–T3: 6.1%) subgroups, but very few to the DP (T1–T2: 1.4%, T2–T3: 0.7%). For the internalizing subgroup, most children transitioned to the no problems subgroup (T1–T2: 43.1%, T2–T3: 51.0%), with some transitioning to the externalizing (T1–T2: 3.4%, T2–T3: 7.7%) and DP (T1–T2: 15.5%, T2–T3: 3.7%) subgroups. Those children in the externalizing subgroup were also likely to transition to the no problems subgroup (T1–T2: 35.6%, T2–T3: 33.4%), and to a lesser extent to the internalizing (T1–T2: 10.1%, T2–T3: 8.1%) and DP (T1–T2: 17.0%, T2–T3: 10.7%) subgroups. Lastly, for those in the DP, the transition probability towards the no problems group decreased with age (T1–T2: 21.9%, T2–T3: 8.6%), whereas the probability of transitioning towards the internalizing subgroup (T1–T2: 11.1%, T2–T3: 35.6%) increased with age and transitioning to the externalizing subgroup remained stable across age (T1–T2: 22.3%, T2–T3: 22.1%). Transition probabilities for the model including covariates were similar to those obtained in the model without covariates and are presented in Supplementary Table 5 and 6.

## Discussion

We utilized three time points of behavioral assessments in a large, population-based birth cohort to examine stability and change in symptoms of psychopathology from childhood into adolescence. Similar to earlier studies, subgroups of psychopathology included four profiles, namely; no problems, internalizing, externalizing and DP [6, 7, 13]. In line with our hypotheses, we observed that externalizing behavior becomes more stable with age, but that the stability of children remaining within the DP subgroup decreases from late childhood into adolescence. Contrary to our hypothesis, internalizing problems did not become increasingly stable with age. Interestingly, the majority (51%) of the children classified in the internalizing subgroup in late childhood progressed to the no problems group in adolescence. Our most notable finding is that, while there is considerable change in behavior for children classified in the DP in childhood, the vast majority (91% of the children) remain in one of the three psychopathology subgroups.

Not surprising, the largest number of children was classified as having no problems across childhood and adolescence. Further, the homotypic continuity observed in this subgroup was 85–88%, meaning that most individuals that do not exhibit psychopathology at an early age, will remain having no psychopathology in adolescence. However, our results also indicate that 5–8% of the children that had no problems in either early or late childhood, transition towards the internalizing group in early childhood or early adolescence, and similarly about 6% transitioned towards the externalizing subgroup. Studying potential underlying mechanisms, including genetic, biological and environmental factors, could ultimately help study and implement prevention strategies targeted on those individuals at-risk.

Together with the work by Basten et al. we show that from late toddlerhood until late childhood the percentage of children included in the internalizing subgroup increases, whereas the rates of children classified in the internalizing subgroup remains stable from late childhood into adolescence [6]. Indeed, studies assessing the age of onset for internalizing disorders find that anxiety disorders, dependent of the subtype of disorder, can emerge at any time during life, whereas the incidence of mood disorders begins to rise during adolescence [15]. However, the median age of onset for specific anxiety and mood disorders is either in early childhood or after early adolescence, with the exception of social phobia. The stable prevalence we observe between late childhood and early adolescence suggests a certain stability in the rate of anxiety symptoms, with fewer children developing internalizing symptoms during this age range. More importantly, in late childhood and early adolescence we found that an increasing proportion of those who transition to the internalizing symptoms subgroup, transitioned either from the externalizing or the DP subgroup at an earlier time point. Thus, these individuals represent a group of children who are already identifiably at-risk, as opposed to those who develop internalizing symptoms after initially having no problems. A future extension of our findings should assess whether intervening to reduce externalizing and DP symptoms can help prevent the later development of internalizing symptoms in those individuals.

We found an increase in the percentage of children with externalizing behavior as these children develop from early childhood into adolescence. This pattern has been observed in earlier work using LPA [7] and it is known that externalizing disorders, such as oppositional defiant disorder and conduct disorder, can develop until late childhood [15, 24, 25]. However, studies have also found a decrease in continuous externalizing symptoms using growth modelling in this age range [26]. While at first glance these results appear contradicting, LTA results obtained in our study as well as in earlier work [7] show that children transitioning to the externalizing subgroup are mainly those who were in the DP at earlier time points. As those in the DP have higher symptoms on all syndrome scales, this increase in individuals in the externalizing subgroup is in line with a decrease in continuous externalizing symptoms. Moreover, those children who transitioned out of the externalizing subgroup, transitioned primarily to the no problems subgroup, which also translates into an overall decrease in externalizing symptoms.

Although a similar pattern for stability within the internalizing and externalizing subgroups was observed earlier, the homotypic continuity we observe is lower than previously reported [7]. Most notably, a larger number of children from the internalizing and externalizing subgroups in early or late childhood transitioned to the no problems group over time, suggesting that for many children symptoms of psychopathology during childhood are a transient phase of development. The differences in homotypic continuity might reflect actual differences between the samples used, but are also likely to be partially dependent on the differences in how psychopathology is reported on the CBCL used here and the Development and Wellbeing Assessment (DAWBA) used in earlier work [27]. Whereas the CBCL is a continuous measure of psychopathology, the DAWBA is a structured clinical interview to diagnose psychopathology. Because of our use of continuous measures, the internalizing and externalizing subgroup consists of children with mostly subclinical symptoms. Possibly, homotypic continuity is higher for those with clinical diagnoses than for those with subclinical symptoms. It would thus be interesting to study whether the initial level of symptoms is predictive of the likelihood that children have persistent problems in a sample enriched for children with subclinical and clinical levels of psychopathology.

Consistent with earlier literature and the conceptualization of the DP, we found that the prevalence of the dysregulation profile is highest in late childhood, after which there is a decline into adolescence [28]. Regarding the development of DP symptoms, we show that those children who already exhibit internalizing or externalizing symptoms are most likely to transition to the DP. However, similar to earlier work, and in line with decreasing prevalence of DP with age, few children transition to the DP after late childhood. Those who are in the DP in early adolescence largely originated from the externalizing and DP subgroups. Notably, the homotypic continuity of the DP decreases between late childhood and early adolescence. Together with the decrease in prevalence, this implies that, for most individuals, psychopathology becomes more clustered within the internalizing or externalizing domain. Despite this decrease in homotypic continuity, our results support the evidence that the DP is an at-risk state for persistent psychopathology, in which the risk of persistence increases with the age at which the DP is exhibited [2, 3, 5]. Where between early and late childhood, 23% of those children that are in the DP transitioned to the no problems group, only 9% of children in the DP transitioned to the no problems group between late childhood and early adolescence. Thus, children exhibiting DP symptoms in late childhood are an optimal target for future intervention studies, as those children are likely to benefit most from early treatment.

The strengths of our study include the large sample size and the longitudinal design embedded within a population-based cohort. Moreover, using data-driven approaches, namely LPA and LTA, we were able to separately measure psychopathology in a more integrated way, compared to assessing individual traits that are likely correlated. Despite these strengths, this study should be considered in light of some limitations. First, we included parental report of child behavior only, other informants (self-report, teacher report) may provide other valuable insights into the development of childhood psychopathology. Unfortunately, we do not have parallel repeated measures of other informants. Second, at T1, the CBCL version 1.5–5 was used, where for those that were older than 5 years of age at assessment, the CBCL version 6–18 would have been more appropriate. At the time of data-collection the decision to use the version 1.5–5 was made to maintain consistency with earlier waves of data-collection not included in the current study. Third, at T2, fit indices provided almost equal support for a four and five profile fit (Supplementary Table 2). However, after visual inspection of the profiles that emerged from our LPA with 5 subgroups, the additional subgroup seemed to be a mix of children with internalizing problems and children with the DP (Supplementary Fig. 1). This was further supported by the results that we obtained from rerunning the LTA with 5 profiles at T2. Most children that were included in the additional fifth profile transitioned to either the internalizing subgroup or the DP at T3 (Supplementary Table 7).

We present both the prevalence and characteristics of psychopathology subgroups and the stability and change that children exhibit in their behavioral development across childhood and adolescence in a large, longitudinal population-based study. Our findings suggest that for many children, internalizing and externalizing problems can be considered a transient phase of development, but that for externalizing problems the predictive value of persistent problems increases with age. Children classified in the DP in late childhood are much more likely to have psychopathology later and the divergence to more specific patterns of psychopathology begins in early adolescence.

## Summary

In summary, we assessed both the stability and nature of change in symptoms of psychopathology from childhood into early adolescence. Using data-driven methods, latent profile analysis (LPA) and latent transition analysis (LTA), we estimated psychopathology subgroups and the transition probabilities between the different psychopathology subgroups and the no problems subgroup. Similar to earlier work both within our sample and in other samples [6, 7, 13], we identified four behavioral subgroups; including ‘no problems’ (T1: 85.9%, T2: 79.0%, T3: 78.0%), ‘internalizing’ (T1: 5.1%, T2: 9.2%, T3: 9.0%), ‘externalizing’ (T1: 7.3%, T2: 8.3%, T3: 10.2%) and a ‘dysregulation’ profile (DP) (T1: 1.7%, T2: 3.5%, T3: 2.8%). Many children who were classified in the internalizing and externalizing subgroup transitioned to the no problems subgroup later in development. However, those classified in the DP in late childhood were likely to have persistent psychopathology, with 91.4% being in either the internalizing, externalizing or DP subgroup in early adolescence. This supports evidence that, for some children, internalizing and externalizing problems can be a transient phase of development, whereas the DP is a severe at-risk state for persistent psychopathology.

## Supplementary Information

Below is the link to the electronic supplementary material.

Supplementary file1 (DOCX 460 kb)

## Data Availability

Generation R data is available to researchers upon reasonable request. Request should be directed to the management team of the Generation R Study. Individual level data are not publicly available for privacy and ethical restrictions.

## Code Availability

The code is available upon request from the corresponding author.
