# Supplementary material for: Stability and Change of Psychopathology Symptoms Throughout Childhood and Adolescence
Source: Child Psychiatry Hum Dev. 2021 Jun 28;53(6):1330–9. doi: 10.1007/s10578-021-01212-8 (PMC9560913; doi:10.1007/s10578-021-01212-8)
Supplement: Supplementary file 1 — Supplementary file1 (DOCX 460 kb) [file 10578_2021_1212_MOESM1_ESM.docx]

**Supplemental material**

| **Supplementary Table 1. Overview of missingness pattern per demographic characteristic** | | | | | | | | |
| --- | --- | --- | --- | --- | --- | --- | --- | --- |
| Demographic Characteristic | Subgroup | Number of participants in subgroup | Number of participants with 1 measurement | Number of participants with 2 measurements | Number of participants with 3 measurements | Percentage of participants with 1 measurement | Percentage of participants with 2 measurements | Percentage of participants with 3 measurements |
| Maternal education | Low | 508 | 257 | 134 | 117 | 50.6% | 26.4% | 23.0% |
|  | Middle | 2698 | 746 | 687 | 1265 | 27.7% | 25.5% | 46.9% |
|  | High | 3200 | 470 | 639 | 2091 | 14.7% | 20.0% | 65.3% |
| Household income | < €2000,- per month | 1351 | 464 | 379 | 508 | 34.3% | 28.1% | 37.6% |
|  | > €2000,- per month | 4493 | 664 | 884 | 2945 | 14.8% | 19.7% | 65.5% |
| National origin | Western | 4741 | 858 | 990 | 2893 | 18.1% | 20.9% | 61.0% |
|  | Non Western | 2103 | 784 | 580 | 739 | 37.3% | 27.6% | 35.1% |

| **Supplementary Table 2. Fit statistics for latent profile models at T1, T2 and T3** | | | | | | | |
| --- | --- | --- | --- | --- | --- | --- | --- |
|  | Number of Profiles | BIC | BLRT | Entropy | Smallest profile |  |  |
| T1 | 2 | 239532.4 | <0.001 | 0.979 | 8.80% |  |  |
|  | 3 | 234820.8 | <0.001 | 0.968 | 2.66% |  |  |
|  | 4 | 231719.7 | <0.001 | 0.982 | 1.73% |  |  |
|  | 5 | 229221.2 | <0.001 | 0.988 | 0.97% |  |  |
|  | 6 | 2270036 | <0.001 | 0.989 | 0.66% |  |  |
| T2 | 2 | 224485.6 | <0.001 | 0.968 | 12.59% |  |  |
|  | 3 | 220740.8 | <0.001 | 0.951 | 3.97% |  |  |
|  | 4 | 218509 | <0.001 | 0.964 | 3.52% |  |  |
|  | 5 | 217158.1 | <0.001 | 0.963 | 1.76% |  |  |
|  | 6 | 216078.3 | <0.001 | 0.966 | 1.11% |  |  |
| T3 | 2 | 217893.2 | <0.001 | 0.957 | 15.56% |  |  |
|  | 3 | 214312.3 | <0.001 | 0.945 | 4.57% |  |  |
|  | 4 | 211789 | <0.001 | 0.967 | 2.83% |  |  |
|  | 5 | 210360.9 | <0.001 | 0.935 | 2.59% |  |  |
|  | 6 | 209497 | <0.001 | 0.957 | 2.78% |  |  |
| BIC = Bayesian information criterion, BLRT = Bootstrapped likelihood-ratio test. | | | | | |  |  |

| **Supplementary Table 3. Stability of psychopathology from T1 to T2 with equal profiles between T2 and T3 (partial invariant model)** | | | | |
| --- | --- | --- | --- | --- |
|  | No problems | Internalizing | Externalizing | Dysregulation |
| No problems | **0.854** | 0.064 | 0.069 | 0.013 |
| Internalizing | 0.441 | **0.382** | 0.040 | 0.137 |
| Externalizing | 0.346 | 0.103 | **0.386** | 0.165 |
| Dysregulation | 0.219 | 0.121 | 0.227 | **0.434** |
| ^1.^ Profiles on the x-axis represent T2, profiles on the y-axis represent T1 | | | | |
| ^2.^ Profiles were held equal between T2 and T3, but not between T1 and T2 due to different versions of the CBCL (at T1: CBCL 1.5-5, at T2 and T3: CBCL 6-18) | | | | |
| ^3.^ Bold numbers indicate homotypic continuity | | | | |
| ^4.^ Model accounted for missing data using full information maximum likelihood (FIML) in Mplus | | | | |

| **Supplementary Table 4. Stability of psychopathology from T2 to T3 with equal profiles between T2 and T3 (partial invariant model)** | | | | |
| --- | --- | --- | --- | --- |
|  | No problems | Internalizing | Externalizing | Dysregulation |
| No problems | **0.879** | 0.060 | 0.054 | 0.007 |
| Internalizing | 0.443 | **0.434** | 0.061 | 0.062 |
| Externalizing | 0.344 | 0.089 | **0.460** | 0.107 |
| Dysregulation | 0.079 | 0.312 | 0.231 | **0.378** |
| ^1.^ Profiles on the x-axis represent T3, profiles on the y-axis represent T2 | | | | |
| ^2.^ Profiles were held equal between T2 and T3, but not between T1 and T2 due to different versions of the CBCL (at T1: CBCL 1.5-5, at T2 and T3: CBCL 6-18) | | | | |
| ^3.^ Bold numbers indicate homotypic continuity | | | | |
| ^4.^ Model accounted for missing data using full information maximum likelihood (FIML) in Mplus | | | | |

| **Supplementary Table 5. Stability of psychopathology from T1 to T2 with correction for covariates** | | | | |
| --- | --- | --- | --- | --- |
|  | No problems | Internalizing | Externalizing | Dysregulation |
| No problems | **0.847** | 0.083 | 0.060 | 0.011 |
| Internalizing | 0.448 | **0.384** | 0.041 | 0.127 |
| Externalizing | 0.375 | 0.093 | **0.373** | 0.159 |
| Dysregulation | 0.201 | 0.111 | 0.254 | **0.434** |
| ^1.^ Profiles on the x-axis represent T2, profiles on the y-axis represent T1 | | | | |
| ^2.^ Profiles were not held equal over time, due to the fact that different versions of the CBCL were used (at T1: CBCL 1.5-5, at T2 and T3: CBCL 6-18) | | | | |
| ^3.^ Bold numbers indicate homotypic continuity | | | | |
| ^4.^ Model accounted for missing data using full information maximum likelihood (FIML) in Mplus | | | | |

| **Supplementary Table 6. Stability of psychopathology from T2 to T3 with correction for covariates** | | | | |
| --- | --- | --- | --- | --- |
|  | No problems | Internalizing | Externalizing | Dysregulation |
| No problems | **0.879** | 0.053 | 0.059 | 0.008 |
| Internalizing | 0.510 | **0.350** | 0.062 | 0.078 |
| Externalizing | 0.309 | 0.052 | **0.547** | 0.092 |
| Dysregulation | 0.071 | 0.265 | 0.251 | **0.413** |
| ^1.^ Profiles on the x-axis represent T3, profiles on the y-axis represent T2 | | | | |
| ^2.^ Profiles were not held equal over time, due to the fact that different versions of the CBCL were used (at T1: CBCL 1.5-5, at T2 and T3: CBCL 6-18) | | | | |
| ^3.^ Bold numbers indicate homotypic continuity | | | | |
| ^4.^ Model accounted for missing data using full information maximum likelihood (FIML) in Mplus | | | | |

| **Supplementary Table 7. Stability of psychopathology from T2 to T3 (five subgroups at T2)** | | | | |
| --- | --- | --- | --- | --- |
|  | No problems | Internalizing | Externalizing | Dysregulation |
| No problems | **0.887** | 0.045 | 0.061 | 0.007 |
| Internalizing | 0.616 | **0.269** | 0.088 | 0.026 |
| Externalizing | 0.334 | 0.079 | **0.487** | 0.101 |
| Internalizing & Dysregulation | 0.259 | **0.535** | 0.088 | **0.117** |
| Dysregulation | 0.067 | 0.205 | 0.255 | **0.479** |
| ^1.^ Profiles on the x-axis represent T3, profiles on the y-axis represent T2 | | | | |
| ^2.^ Profiles were not held equal over time, due to the fact that different versions of the CBCL were used (at T1: CBCL 1.5-5, at T2 and T3: CBCL 6-18)  ^3.^ Bold numbers indicate homotypic continuity | | | | |
| ^4.^ Model accounted for missing data using full information maximum likelihood (FIML) in Mplus | | | | |

**Supplementary Figures**

**
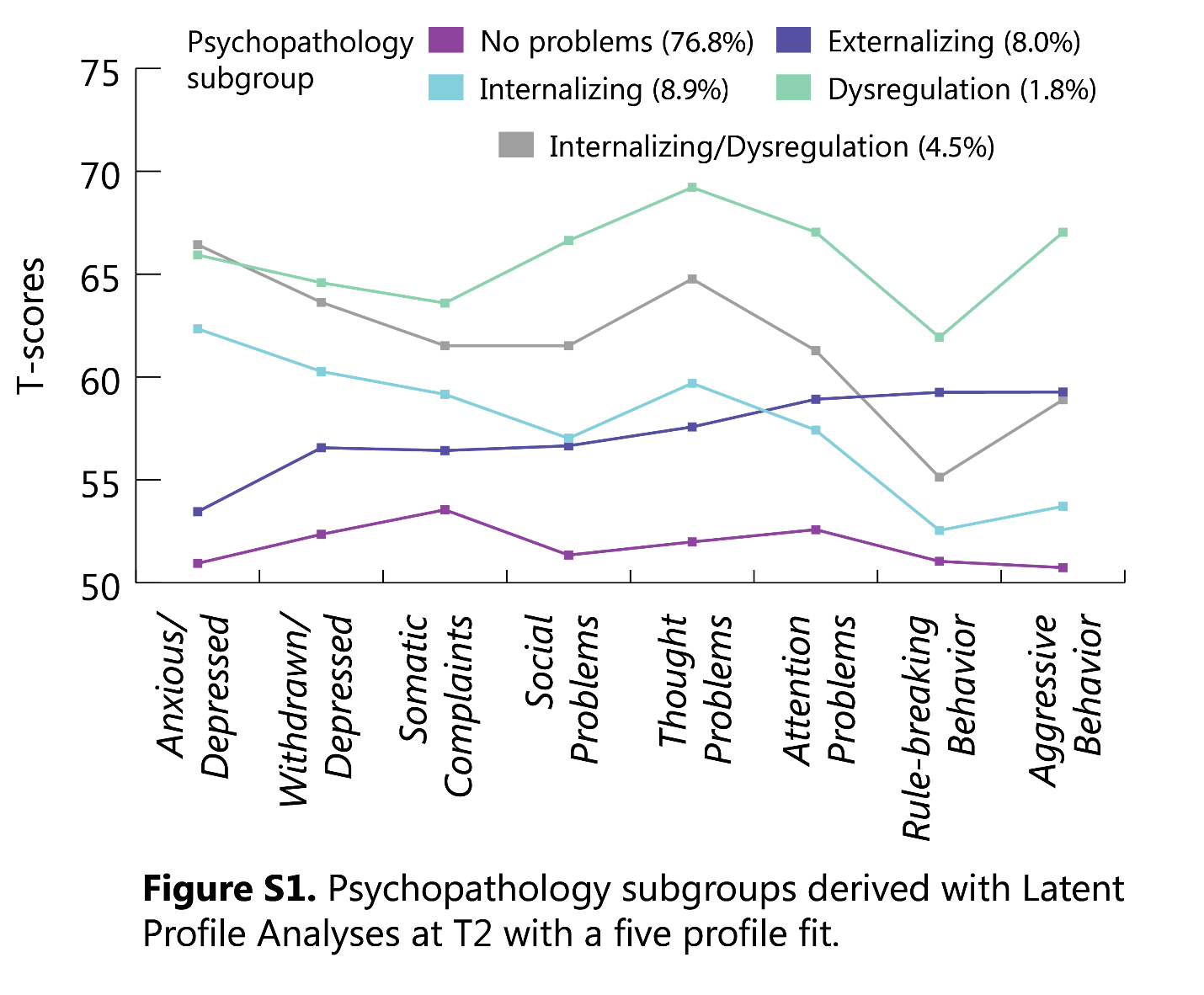
**

**
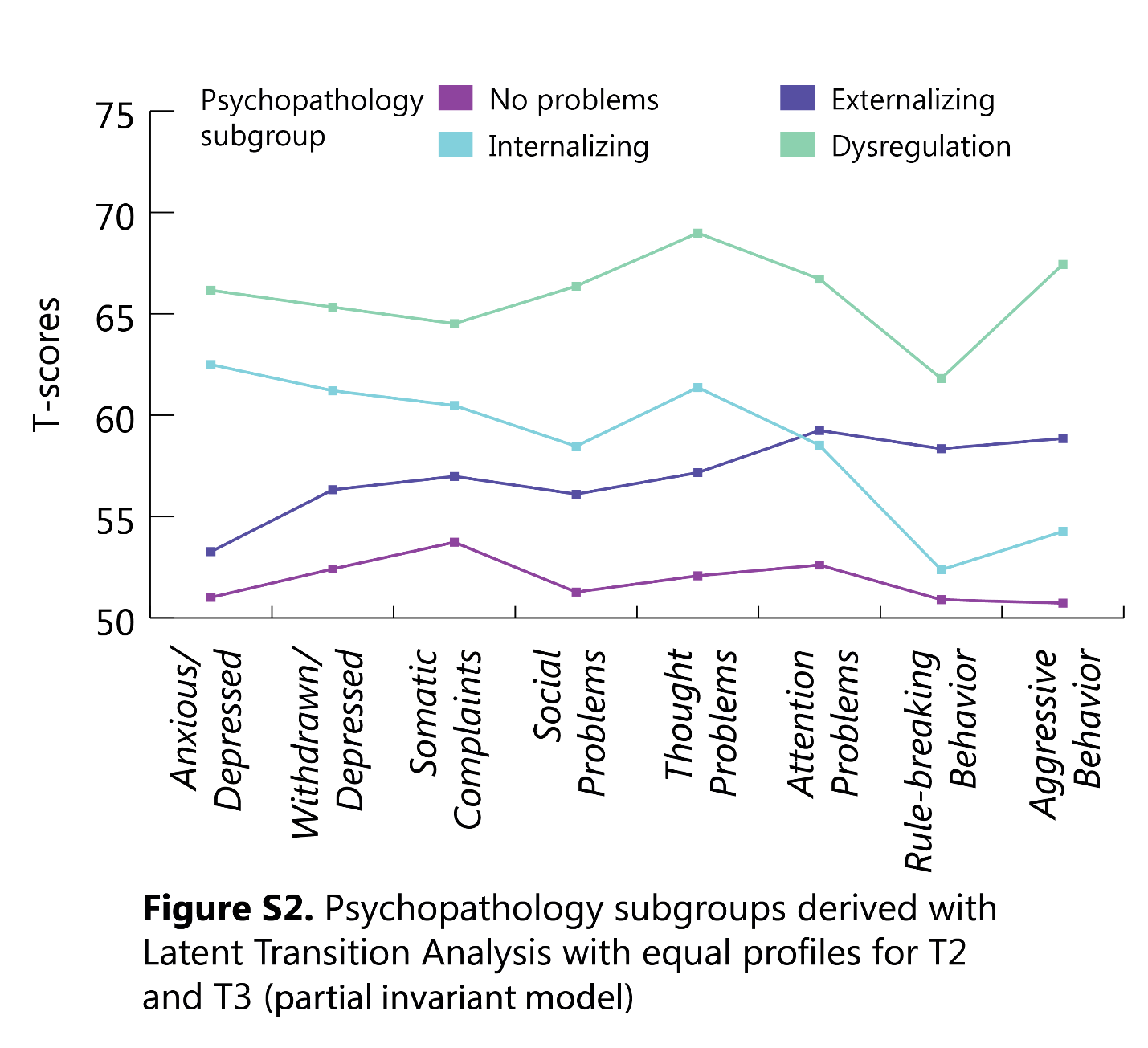
**
